# Novel Picobirnaviruses in Respiratory and Alimentary Tracts of Cattle and Monkeys with Large Intra- and Inter-Host Diversity

**DOI:** 10.3390/v11060574

**Published:** 2019-06-23

**Authors:** Patrick C. Y. Woo, Jade L. L. Teng, Ru Bai, Ying Tang, Annette Y. P. Wong, Kenneth S. M. Li, Carol S. F. Lam, Rachel Y. Y. Fan, Susanna K. P. Lau, Kwok-Yung Yuen

**Affiliations:** 1 State Key Laboratory of Emerging Infectious Diseases, The University of Hong Kong, Hong Kong, China; llteng@hku.hk (J.L.L.T.); kyyuen@hku.hk (K.-Y.Y.); 2 Department of Microbiology, The University of Hong Kong, Hong Kong, China; fionabai314@gmail.com (R.B.); ytang.ashley@gmail.com (Y.T.); annettewongyp@gmail.com (A.Y.P.W.); keth105@gmail.com (K.S.M.L.); carollamsukfun@yahoo.com.hk (C.S.F.L.); rachelfyy2004@yahoo.com.hk (R.Y.Y.F.); 3 Carol Yu Centre for Infection, The University of Hong Kong, Hong Kong, China; 4 Collaborative Innovation Center for Diagnosis and Treatment of Infectious Diseases, The University of Hong Kong, Hong Kong, China

**Keywords:** novel picobirnaviruses, respiratory tracts, cattle, monkeys, diversity

## Abstract

Picobirnaviruses (PBVs) are mostly found in animal alimentary samples. In this study, among 576 respiratory specimens from 476 mammals and 100 chickens, genogroup I PBVs were detected in three cattle and three monkeys, and a genogroup II PBV-positive sample was collected from one cattle specimen. More than one PBV sequence type was observed in two and one genogroup I PBV-positive samples from cattle and monkeys, respectively. Twenty-four complete/near-complete segments 2 (nine from respiratory and 15 from alimentary samples) from the cattle and monkey genogroup I PBVs and one complete segment 2 from the cattle genogroup II PBV were sequenced. Similar to other studies, the cattle PBVs also showed a high diversity. In contrast, the monkey PBVs observed in this study were clustered into three distinct clades. Within each clade, all the sequences showed >99% amino acid identities. This unique phenomenon is probably due to the fact that monkeys in our locality reside in separated troops with minimal inter-troop contact.

## 1. Introduction

Picobirnaviruses (PBVs) are small non-enveloped bi-segmented double-stranded RNA viruses. Since their first discovery in alimentary samples of humans and rats in 1988 [1,2], PBVs have been reported in other terrestrial and marine mammals, birds, invertebrates and environmental water samples [3,4,5,6,7,8,9,10,11,12,13,14,15,16,17,18,19]. Recently, we have described the first discovery and a high diversity of PBVs in California sea lions from an oceanarium in Hong Kong and dromedary camels from the Middle East [18,20,21]. We also found that multiple strains of PBV were present in the majority of PBV-positive samples in different kinds of animals [18].

The genome of a PBV consists of two segments: segment 1 and segment 2. Segment 1 contains the capsid gene and another open reading frame which encodes a putative protein of an unknown function, whereas segment 2 contains the RNA-dependent RNA polymerase (RdRp) gene [22]. By conducting sequence and phylogenetic analyses of RdRp genes, PBVs were classified into genogroups I and II [22,23]. Recently, it was reported that a third genogroup of PBV, genogroup III, was obtained from invertebrate hosts and differs largely from the PBVs of genogroups I and II [15,24]. In our recent study in which serial samples from the same animal were collected, we observed that PBV probably evolves through mechanisms similar to other segmented RNA viruses, such as rotaviruses (double-stranded RNA virus) [18]. Rotaviruses evolve through reassortment of their genomes, resulting in enormous diversity [25,26]. This also contributes to the emergence of new rotavirus strains, resulting in disease outbreaks [25,26]. However, in a recent study on PBV detected in a marmot of the Qinghai-Tibet plateau, it was found that segmentation-associated motifs were identified in both bi-segmented and unsegmented PBVs. Unsegmented PBVs may undergo genome segmentation at the motif region and convert to segmented PBVs, which represents a unique segmentation mechanism [9]. 

Traditionally, PBVs were found in alimentary samples of animals. However, in two recent studies, PBVs were amplified from >10% and 0.97% of respiratory samples collected from pigs and a human, respectively [27,28]. Such discoveries have improved our understandings on the tissue tropism of PBVs in the corresponding hosts. We hypothesize that PBVs may also reside in the respiratory tracts of animals other than pigs and humans. To test this hypothesis, we carried out a molecular epidemiology study of PBVs in respiratory samples collected from mammals and poultry. Genome segment 2 sequencing, comparative genomics, and phylogenetic analysis of the PBVs detected in the respiratory and alimentary samples of cattle and monkeys were also performed. 

## 2. Materials and Methods 

### 2.1. Ethics Statement

The study was approved by the Committee on the Use of Live Animals for Teaching and Research, The University of Hong Kong (CULATR Ref. No.: 2284-10 and 3330-14; Date of approval: 23 March 2011 and 17 April 2014). 

### 2.2. Mammal and Poultry Surveillance and Sample Collection

All specimens of bats, monkeys, dogs, and cats were collected with the assistance of the Department of Agriculture, Fisheries and Conservation (AFCD), Hong Kong Special Administrative Region (HKSAR); those of cattle and chickens were collected with the assistance of the Department of Food, Environmental and Hygiene (FEHD), HKSAR, from various locations in HKSAR; and those of horses were collected with the assistance of the Hong Kong Jockey Club. All respiratory specimens (nasopharyngeal swabs in cattle, dogs, cats, and horses; throat swabs in monkeys; mouth swabs in bats; and tracheal swabs in chickens) were collected using procedures described previously [29]. A total of 576 samples collected over an 83-month period (March 2006 to January 2013) in Hong Kong from 157 bats, 52 monkeys, 50 dogs, 50 cats, 116 horses, 51 cattle, and 100 chickens have been tested (Table 1). 

### 2.3. RNA Extraction

Viral RNA was extracted from the respiratory samples using the EZ1 Virus Mini Kit v2.0 according to the manufacturer’s instructions (Qiagen, Hilden, Germany). The RNA was eluted in 60 μL of AVE buffer (Qiagen, Hilden, Germany) and used as the template for RT-PCR.

### 2.4. RT-PCR for PBVs and DNA Sequencing

PBV screening was performed by PCR amplification of a 205-bp (nucleotide position 967–1171) and a 207-bp fragment (nucleotide position 786–992) of the RdRp gene of genogroup I and genogroup II PBVs, respectively, using conserved primers (genogroup I: 5′-CAAARTTYGACCARCACTT-3′ and 5′-TCRTCDGCRTTGGTACCACC-3′; genogroup II: 5′-WTGGATGTTTCCGATGTC-3′ and 5′-TGYGCATCCATYTTMGTGGTGTCTC-3′) designed based on multiple alignments of the nucleotide sequences of available RdRp genes of PBVs [30]. Reverse transcription was performed using the SuperScript III kit (Invitrogen, San Diego, CA, USA) and the reaction mixture (10 μL) contained RNA, first-strand buffer (50 mM Tris-HCl pH 8.3, 75 mM KCl, 3 mM MgCl_2_), 5 mM dithiothreitol, 50 ng random hexamers, 500 μM of each deoxyribonucleotide triphosphate and 100 U Superscript III reverse transcriptase. The mixtures were incubated at 25 °C for 5 min, followed by 50 °C for 60 min and 70 °C for 15 min. The PCR mixture (25 μL) contained cDNA, PCR buffer (10 mM Tris-HCl pH 8.3, 50 mM KCl, 2 mM MgCl_2_), 200 μM of each dNTP, 10 μM of each primer, and 1.0 U *Taq* polymerase (Applied Biosystems, Foster City, CA, USA). The mixtures were amplified in 60 cycles of 94 °C for 1 min, 50 °C for 1 min, and 72 °C for 1 min, and a final extension at 72 °C for 10 min in an automated thermal cycler (Applied Biosystems, Foster City, CA, USA). Standard precautions were taken to avoid PCR contamination and no false-positive was observed in the negative controls.

All PCR products were gel-purified using the QIAquick gel extraction kit according to the manufacturer’s instructions (Qiagen, Hilden, Germany). Both strands of the PCR products were sequenced twice with a Prism 3700 DNA analyzer (Applied Biosystems, Foster City, CA, USA), using the two PCR primers. When multiple nucleotide peaks were observed in the sequencing results, which suggested more than one type of PBV in the sample, the corresponding purified PCR product was cloned into the pCR-II-TOPO TA vector (Invitrogen, San Diego, CA, USA) according to the manufacturer’s instructions. Both strands of 10 clones for each sample were sequenced, using primers 5′-TAATACGACTCACTATAGGG-3′ and 5′-CGGCTCGTATGTTGTGTGGA-3′, which bind to the nucleotide position 160–179 and 406–425 of the vector, respectively. The sequences of the PCR products or the clones were compared with known sequences of the RdRp of PBVs in the GenBank database.

### 2.5. Complete/Near-Complete Segment 2 Sequencing of Genogroups I and II PBVs

Five and four complete segment 2 samples of cattle and monkey genogroup I PBVs, and one complete segment 2 of cattle a genogroup II PBV detected in the respiratory specimens were amplified and sequenced respectively using published strategies for double-stranded RNA viruses [31]. In addition, six and nine complete/near-complete segment 2 samples of the cattle and monkey PBVs we previously detected in the alimentary specimens were also amplified and sequenced using the same strategy [31]. RNA extracted from the original respiratory and alimentary specimens, using the EZ1 virus mini kit (Qiagen, Hilden, Germany), was used as the template for RT-PCR and sequencing. An adaptor primer, with a 3′ NH_2_ blocking group, was ligated to the 3′ termini of the viral RNA and subjected to reverse transcription using a complementary primer. After RNA hydrolysis, reannealing, and end-filling, single-primer amplification of viral genomic segments was performed using a complementary primer and genome specific primers using a strategy described previously [32]. The 5′ and 3′ ends of the viral genomes were confirmed by the rapid amplification of cDNA ends using the 5′/3′ RACE kit (Clontech, Mountain View, CA, USA). The PCR products were gel purified and cloned into the pCR-Blunt II-TOPO vector by employing the Zero Blunt TOPO PCR cloning kit (Invitrogen, San Diego, CA, USA). Clones were sequenced using an ABI Prism 3700 DNA analyzer (Applied Biosystems, Foster City, CA, USA). Sequences were assembled and manually edited to produce the final sequences of the segment 2 genome. 

### 2.6. Genome Segment 2 Analysis

The nucleotide sequences of genome segment 2 and the deduced amino acid sequences of the open reading frames (ORFs) were compared to those of other PBVs. Phylogenetic analysis was performed by the neighbor-joining method, using the Jukes–Cantor substitution model with gamma distributed rate variation and bootstrap values calculated from 1000 trees [30]. A clade is defined as two or more PBVs sharing >99% amino acid identities in their complete or near-complete RdRp sequences. Secondary structure prediction in the 5′UTR was performed using RNAfold [33].

### 2.7. Estimation of Synonymous and Non-Synonymous Substitution Rates

The number of synonymous substitutions per synonymous site, *Ks*, and the number of non-synonymous substitutions per non-synonymous site, *Ka*, for the RdRp between monkey PBVs belonging to clade M-R1 were calculated using the Nei-Gojobori method (Jukes-Cantor) in MEGA6 [30]. The *Ks* and *Ka* for the other two clades of monkey PBVs were not calculated because the number of sequences was small. 

### 2.8. Nucleotide Sequence Accession Numbers

The genome segment 2 sequences of all cattle and monkey PBVs have been lodged within the GenBank sequence database under accession no. KY120170-KY120194.

## 3. Results

### 3.1. Detection of PBVs in Respiratory Specimens of Animals

**Figure 1:**
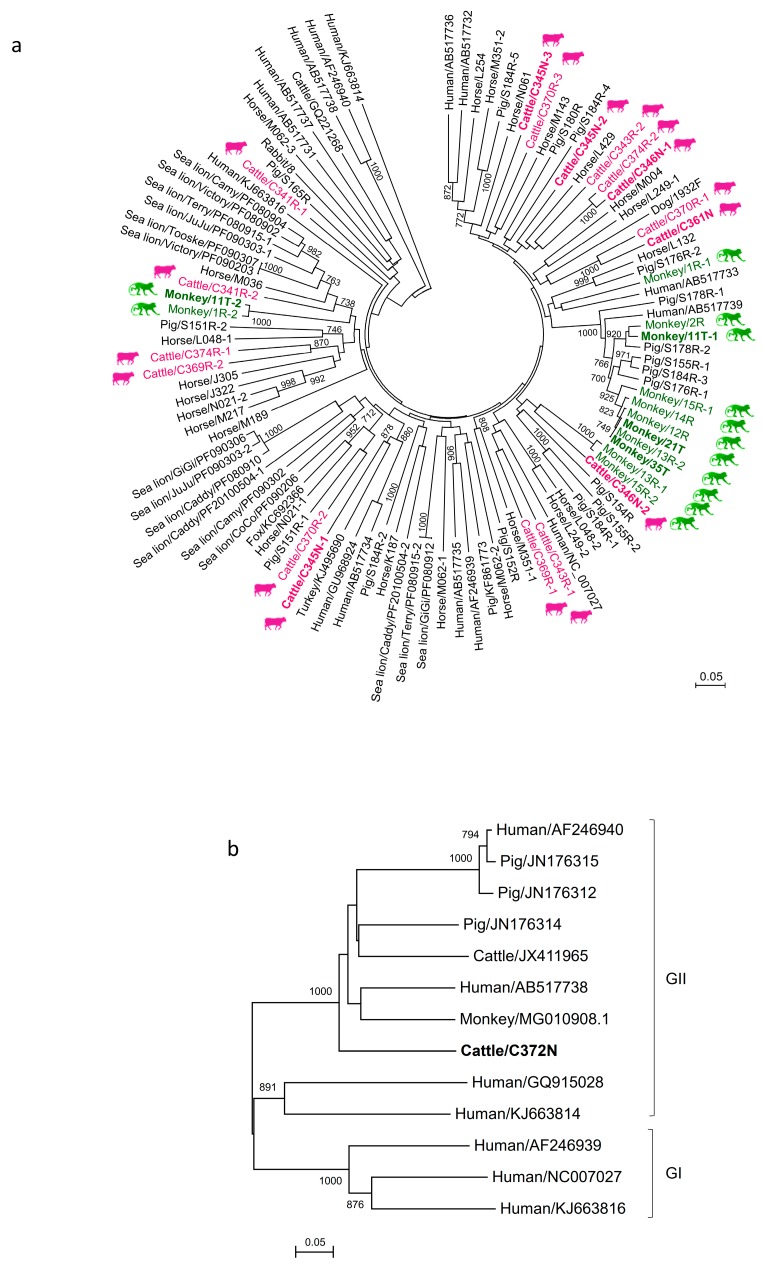
Phylogenetic analysis of a partial RdRp gene of genogroup I and II picobirnaviruses (PBVs) discovered in the present study. The trees were constructed using the neighbor-joining method. Bootstrap values below 70% are not shown. The scale bar indicates the number of nucleotide substitutions per site. All the accession numbers are given as cited in GenBank. (**a**) One hundred and ninety-one nucleotide positions were included in the analysis and rooted with genogroup II human strains, shown in italics. All PBV strains detected in the same host species were highlighted in the same color, with the highlights in bold representing the PBVs discovered in respiratory specimens. If more than one sequence type was found in the same sample, each sequence type was numbered in the order of identification (e.g., Monkey/11T-1 and Monkey/11T-2 indicated that there were two sequence types found in the same monkey sample 11T). (**b**) One hundred and ninety-two nucleotide positions were included in the analysis and rooted with genogroup I human PBV strains. The genogroup II PBV characterized in this study is shown in bold.

A total of 576 respiratory specimens from 476 mammals and 100 chickens were obtained (Table 1). RT-PCR for a 205-bp fragment in the RdRp of a genogroup I PBV was positive in respiratory samples collected from three cattle and three monkeys, respectively (Table 1). Sequencing and phylogenetic analysis showed that the three positive samples from cattle (C345N, C346N, and C361N) contained three (C345N-1, C345N-2, and C345N-3), two (C346N-1 and C346N-2), and one (C361N) sequence types, respectively (Figure 1A). There were 63 (31.3%) to 73 (35.8%) nucleotide differences among C345N-1, C345N-2, and C345N-3 and 62 (30.5%) nucleotide differences between C346N-1 and C346N-2. For the three positive samples from monkeys (11T, 21T, and 35T), two sequence types (11T-1 and 11T-2) were observed in one sample (11T) and one sequence type was observed in the other two samples (21T and 35T) (Figure 1A). There were 64 (31.5%) nucleotide differences between 11T-1 and 11T-2. The PBV sequences from cattle and monkeys were widely distributed in the whole phylogenetic tree with a high diversity (Figure 1A). No genogroup I PBV was amplified from respiratory samples collected from 50 dogs, 50 cats, 157 bats, 116 horses, and 100 chickens. 

RT-PCR for a 207-bp fragment in the RdRp of a genogroup II PBV was positive in one respiratory sample collected from cattle (Table 1). Sequencing and phylogenetic analysis showed that the sequence possessed 46–89 (22.8–50.6%) nucleotide differences to other genogroup II PBV sequences, being most closely related to macaque PBV (MG010908.1) (Figure 1B). No genogroup II PBV was amplified from respiratory samples collected from the 52 monkeys, 50 dogs, 50 cats, 157 bats, 116 horses, and 100 chickens. No co-infection with both genogroups of PBVs in a single sample was observed.

### 3.2. Complete/Near-Complete Segment 2 Sequence Analysis of Cattle and Monkey PBVs

**Figure 2:**
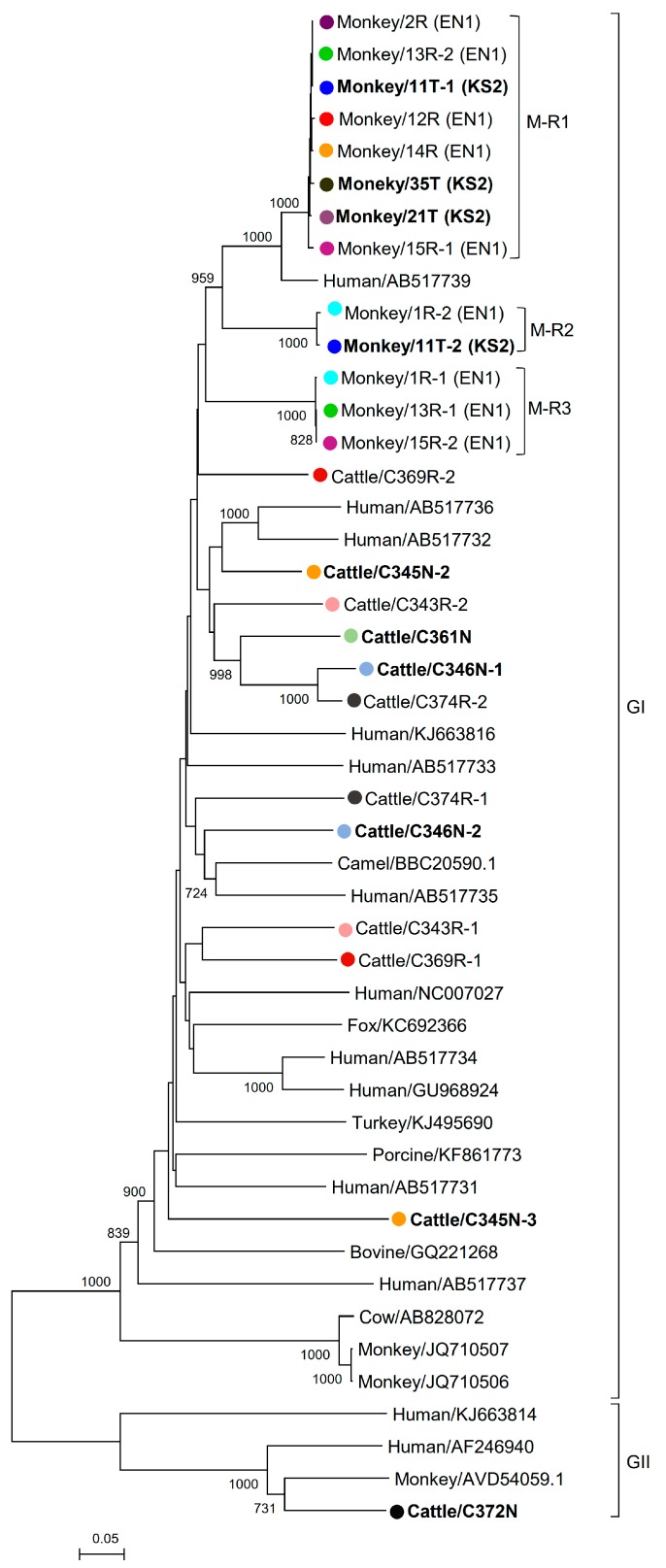
Phylogenetic analysis of RdRp proteins of cattle and monkey picobirnaviruses (PBVs). The trees were constructed using the neighbor-joining method. Four hundred and seventy-five amino acid positions were included in the analysis. The scale bar indicates 0.05 substitutions per site. Bootstrap values below 70% are not shown. All PBV strains detected in the same host were highlighted with the solid dots/circles in the same color, with the highlights in bold representing the PBVs discovered in respiratory specimens. If more than one sequence type was found in the same sample, each sequence type was numbered in the order of identification (e.g., monkey/11T-1 and monkey/11T-2 indicated that there were two sequence types found in the same monkey sample 11T). The clade of each monkey PBV and their sampling locations (EN1: Lion Rock Site 1 and KS2: Kam Shan Site 2) are also indicated. All the accession numbers are given as cited in GenBank. GI and GII represent genogroup I and genogroup II PBVs, respectively.

Twenty complete and four near-complete segment 2 samples (nine from respiratory and 15 from alimentary samples) from the cattle and monkey genogroup I PBVs were successfully sequenced and assembled (Table 2, Figure 2). For the three positive alimentary samples from cattle (C343R, C369R, and C374R), each of them contained two sequence types (C343R-1 and C343R-2, C369R-1 and C369R-2, and C374R-1 and C374R-2, respectively). For the six positive alimentary samples from monkeys (1R, 2R, 12R, 13R, 14R, and 15R), two sequence types (1R-1 and 1R-2, 13R-1 and 13R-2, and 15R-1 and 15R-2, respectively) were observed in three samples (1R, 13R, and 15R) and one sequence type was observed in the other three samples (2R, 12R, and 14R) [18]. Sequencing of the other eight segment 2 samples was not successful because of low viral loads and/or failure to amplify part of the segment. These 24 sequenced segment 2 samples ranged from 1613 to 1736 bases in length, with overall G+C contents of 41.1 to 47.7%. The 5′ non-coding regions (27 to 74 bases) are AU-rich (G+C contents 14.8 to 35.4%) with five conserved bases, GUAAA, located at the 5′ end. A highly stable stem loop structure observed in other known PBVs was observed in 17 of 20 complete segment 2 sequences as a result of the pairing of 5′-GUAAA-3′ and 5′-UUUAC-3′ sequences in the 5′ non-coding region [34]. The 3′ non-coding regions (15 to 50 bases) had G+C contents that ranged from 40.0 to 59.5%, ending with four conserved bases (CUGC) in most of the genomes. Excluding the one detected in monkey PBV 1R-1 with incomplete RdRp, all the other 23 sequenced segment 2 samples possessed one long ORF (1563 to 1686 bp) encoding the putative RdRp of 520 to 561 amino acids. These putative RdRp sequences shared 43.0 to 80.0% amino acid identities with those of other genogroup I PBV strains, being most closely related to dromedary picobirnavirus (GenBank number BBC20590.1), human PBV HuPBV-E-CDC16 (Genbank number KJ663816), human PBV GPBV6C1 (Genbank number AB517731), human PBV VS10 (Genbank number GU968924), and fox PBV F5-1 (Genbank number KC692366). All segment 2 samples, except the one detected in cattle (C374R-1) with the first motif being D-E-D instead of D-T/S-D, possessed three conserved motifs (D-T/S-D, SG-T, and GDD) commonly found in the RdRp sequences of other dsRNA viruses [6]. Conserved cysteine and proline residues present in other genogroup I PBVs were also observed in all 24 segment 2 sequences [6].

One complete segment 2 from the cattle genogroup II PBV was sequenced and assembled (Table 2, Figure 2). This segment 2 was 1622 bases in length with a G+C content of 40.4%. The 5′ non-coding region (54 bases) was AU-rich (G+C content 16.7%) with the five conserved bases, GUAAA, located at the 5′ end. Similar to genogroup I PBVs, a highly stable stem loop structure was also observed in the 5′ non-coding region as a result of the pairing of 5′-GUAAA-3′ and 5′-UUUAC-3′ sequences [34]. The 3′ non-coding region (23 bases) had a G+C content of 52.2%, ending with CUCC. This segment 2 possessed one long ORF (1545 bp) encoding the putative RdRp of 514 amino acids. This putative RdRp shared 40.7 to 78.0% amino acid identities with those of other genogroup II PBV strains, being most closely related to macaque PBV (Genbank number AVD54059.1). The three conserved motifs (D-T/S-D, SG-T, and GDD) commonly found in the RdRp sequences of other dsRNA viruses were also observed [34].

### 3.3. Phylogenetic Analysis of Cattle and Monkey PBVs’ Complete/Near-Complete Segment 2 Sequences

In the phylogenetic tree constructed using the RdRp gene, the 13 segment 2 sequences of the monkey PBVs were clustered into three (M-R1 to M-R3) distinct clades (Figure 2). Within each clade, all the sequences showed >99% amino acid identities, suggesting that they belong to the same clone. The alimentary samples of three monkeys (1R, 13R, and 15R) possessed sequences that belonged to two different clades (M-R2 and M-R3 for sample 1R; M-R1 and M-R3 for samples 13R and 15R), and the throat swab of one monkey (11T) possessed sequences that belonged to two different clades (M-R1 and M-R2). As for the sequences from the cattle samples, except for two sequences (C346N-1 and C374R-2), no distinct clades were observed, suggesting that there was no dominant clone in these alimentary and respiratory samples (Figure 2). 

### 3.4. Estimation of Substitution Rates

The *Ka, Ks*, and *Ka/Ks* of the RdRp in monkey PBVs belonging to clade M-R1 are 0.002, 0.049 and 0.041, respectively. 

## 4. Discussion

We discovered PBVs from respiratory samples of cattle and monkeys. The original discovery of PBV from alimentary samples raised the question of whether PBV is associated with gastroenteritis [1,2]. The recent findings on the presence of genogroup I and II PBV in respiratory tract swab specimens of pigs, genogroup I PBV in bronchoalveolar lavage of humans, and non-genogroup I/II PBV in nasal swabs of young dairy cattle with cattle respiratory disease further prompted us to rethink whether these viruses could be respiratory tract pathogens [27,28,35]. Moreover, the high abundance of PBV in respiratory samples of pigs is also in line with its high abundance in their alimentary samples [27,36]. In this study, we further provided evidence that PBVs can be detected in respiratory samples of cattle and monkeys in Hong Kong. Although PBV was not detected in respiratory samples of dogs, cats, bats, and chickens in the present study, this may partly be due to the relatively low abundance of PBV in these animals, as our recent study on mammalian PBVs and our unpublished data on poultries also did not reveal a high abundance of PBV in their alimentary samples. Notably, similar to results in previous reports [37,38], genogroup I PBV sequences are more commonly found than genogroup II PBV sequences in the present study. This may be due to a genuine higher abundance of genogroup I PBV or the use of primers better designed for the amplification of genogroup I than genogroup II PBV as a result of the availability of many more genogroup I PBV sequences in GenBank. In the present and our previous studies [18,20], we found that the topologies of the phylogenetic trees constructed based on the partial RdRp sequences (around 200-bp) and complete RdRp sequences of PBVs were completely concordant (i.e., correct classification of PBVs into genogroup I and genogroup II). This implies that sequencing partial RdRp PCR products is already sufficient for classifying genogroup I and genogroup II PBVs.

Multiple strains of PBV were present in the respiratory samples of monkeys and cattle. Up until early 2019, although PBVs have been found in different animal species [2,3,7,8,9,10,13,17,18,19,20,21,39,40,41,42,43,44,45], more than one strain of PBV has only been described in alimentary samples collected from humans [5,38], pigs [36,39], chickens [14], monkeys [17], and buffalos [43]. In our recent study, multiple strains of PBV were observed in the same alimentary samples of cattle, monkeys, horses, pigs, and California sea lions [18]. In the present study, among the three monkeys and four cattle positive for PBVs in their respiratory samples, more than one strain of PBV was present in two cattle and one monkey, as confirmed by RdRp gene sequencing. This phenomenon of multiple strains of PBV in one respiratory sample has been reported in pigs, from which both genogroups I and II PBVs were observed in the same sample [27]. 

The cattle PBVs observed from the respiratory and alimentary samples showed a high diversity. In our recent study, it was found that the PBV sequences from different kinds of mammals showed a high diversity and phylogenetic analysis revealed that they were widely distributed over the phylogenetic tree [18]. Although genogroup I PBVs have been found in different kinds of animals [2,3,7,8,9,10,13,17,18,19,20,21,39,40,41,42,43,44,45], genogroup II PBVs have only been reported in humans [5,46], monkeys [47], pigs [36], a cattle calf [44], and a cat [19]. In this study, both genogroups I and II PBVs were present in cattle, with the genogroup I cattle PBVs showing a high diversity. Except for the two cattle (C346N-1 and C374R-2) of which the RdRp genes of their PBVs showed 93.6% amino acid identity, all the other cattle PBVs had <80% amino acid identities with each other. In our recent study, we proposed that PBVs evolved through genome reassortment and high mutation rates, which may partly account for the high diversities observed in PBVs from different kinds of animals [18]. Furthermore, a recent study reported that PBVs may infect prokaryotes [47], suggesting that the interplay of PBVs and prokaryotes may also account for the evolution and genetic diversity of PBVs.

In contrast to the high diversities observed in PBVs from cattle, horses, pigs, and California sea lions from the present and our previous study, three distinct PBV clones were found in the monkeys in this study. In Hong Kong, most monkeys in the wild are hybrids of the rhesus macaque and long-tailed macaque. The total number of macaques in Hong Kong is about 1800 to 2000. They live as 30 geographically separated troops. The respiratory and alimentary samples collected in this study were from monkeys that belonged to two separated troops 1.6 km apart. For clades M-R1 and M-R2, it can be observed that the same PBV clone was present in both the respiratory and alimentary samples (Figure 2). This implies that the same PBV can probably infect both the respiratory and gastrointestinal tracts of monkeys. This unique phenomenon observed in these PBVs in monkeys in this study has also given us the opportunity to perform molecular evolution studies for protein-coding genes in monkey PBVs of the M-R1 clade, which showed that the *Ka/Ks* ratio was <0.05, implying that this virus was stably evolving in monkeys. Further studies should be performed to examine if there is any association between PBV and diarrhea in monkeys, as well as their diversity and evolutionary mechanism. 

## Figures and Tables

**Table 1:** Prevalence of picobirnaviruses (PBVs) in respiratory samples of animals by RT-PCR.

Animal Type	Sample Types	No. of Specimens Obtained	No. (%) of Specimens Positive for Genogroup I PBVs	No. (%) of Specimens Positive for Genogroup II PBVs
Mammals				
Cattle	Nasopharyngeal swab	51	3 (5.9%)	1 (1.9%)
Monkey ^1^	Throat swab	52	3 (5.8%)	0 (0%)
Dog	Nasopharyngeal swab	50	0 (0%)	0 (0%)
Cat	Nasopharyngeal swab	50	0 (0%)	0 (0%)
Bat ^2^	Mouth swab	157	0 (0%)	0 (0%)
Horse ^3^	Nasopharyngeal swab	116	0 (0%)	0 (0%)
Poultry				
Chicken	Tracheal swab	100	0 (0%)	0 (0%)

^1^ Most monkeys found in our locality are considered hybrids of *Macaca mulatta* and *Macaca fascicularis*. ^2^ Species included *Hipposideros pomona* (*n* = 103), *Rhinolophus affinis* (*n* = 14), and *Rhinolophus sinicus* (*n* = 40). ^3^ Thoroughbred racehorses stabled at the Hong Kong Jockey Club.

**Table 2:** Comparison of genomic features among monkey and cattle picobirnaviruses (PBVs) and other PBV strains with a complete/near complete segment 2 sequence available.

PBV		Genome Features												
		Size (bp)	G+C Content (%)	ORF Features					5′ UTR Features			3′ UTR Features		
				ORF	Location (nt)	Length (nt)	Length (aa)	Frame	Length (nt)	G+C Content (%)	Beginning Bases	Length (nt)	G+C Content (%)	Ending Bases
**Reference genogroup I PBVs segment 2 sequences**														
Human ^1^		1745	46.36	RdRp	94-1698	1605	534	+1	93	21.51	GTAAA	47	53.19	CTGC
Human ^2^		1696	43.63	RdRp	58-1650	1593	530	+1	57	24.56	GTAAA	46	50.00	CTGC
**Reference genogroup II PBV segment 2 sequence**														
Human ^3^		1674	43.91	RdRp	88-1641	1554	517	+1	87	16.09	GTAAA	33	57.58	CTC
**Genogroup I PBVs detected in this study**														
**PBVs (Clade)**	**GenBank accession no.**													
Monkey/11T-1 (M-R1;)	KY120190	1678	42.55	RdRp	28-1632	1605	534	+1	27	14.81	GTAAA	46	52.17	CTGC
Monkey/11T-2 (M-R2)	KY120191	1681	41.05	RdRp	47-1636	1590	529	+2	46	26.09	GTAAA	45	51.11	CTGC
Monkey/35T (M-R1)	KY120193	1678	42.61	RdRp	28-1632	1605	534	+1	27	14.81	GTAAA	46	52.17	CTGC
Monkey/21T (M-R1)	KY120192	1678	42.49	RdRp	28-1632	1605	534	+1	27	14.81	GTAAA	46	52.17	CTGC
Monkey/1R-1 ^4^ (M-R3)	KY120194	1613	42.72	RdRp	2-1564	1563	520	NA	NA	NA	NA	49	53.06	CTGC
Monkey/1R-2 (M-R2)	KY120182	1681	41.52	RdRp	47-1636	1590	529	+2	46	26.09	GTAAA	45	51.11	CTGC
Monkey/2R (M-R1)	KY120183	1678	42.37	RdRp	28-1632	1605	534	+1	27	14.81	GTAAA	46	50.00	CTGC
Monkey/12R (M-R1)	KY120184	1678	42.61	RdRp	28-1632	1605	534	+1	27	14.81	GTAAA	46	52.17	CTGC
Monkey/13R-1 (M-R3)	KY120185	1734	41.64	RdRp	75-1685	1611	536	+3	74	25.68	GTAAA	49	53.06	CTGC
Monkey/13R-2 (M-R1)	KY120186	1678	42.55	RdRp	28-1632	1605	534	+1	27	14.81	GTAAA	46	52.17	CTGC
Monkey/14R (M-R1)	KY120187	1678	42.55	RdRp	28-1632	1605	534	+1	27	14.81	GTAAA	46	52.17	CTGC
Monkey/15R-1 (M-R1)	KY120188	1678	42.31	RdRp	28-1632	1605	534	+1	27	14.81	GTAAA	46	50.00	CTGC
Monkey/15R-2 (M-R3)	KY120189	1734	41.64	RdRp	75-1685	1611	536	+3	74	25.68	GTAAA	49	53.06	CTGC
Cattle/C345N-2	KY120170	1671	43.93	RdRp	49-1629	1581	526	+1	48	25.00	GTAAA	42	52.38	CATC
Cattle/C345N-3	KY120171	1694	42.15	RdRp	44-1657	1614	537	+2	43	23.26	GTAAA	37	59.46	CTGC
Cattle/C346N-1	KY120172	1701	47.68	RdRp	49-1656	1608	535	+1	48	29.17	GTAAA	45	44.44	CTGC
Cattle/C346N-2	KY120173	1696	44.99	RdRp	52-1647	1596	531	+1	51	27.45	GTAAA	49	46.94	CTGC
Cattle/C361N	KY120174	1696	44.58	RdRp	49-1653	1605	534	+1	48	35.42	GTAAA	43	44.19	CTGC
Cattle/C343R-1	KY120175	1692	44.03	RdRp	50-1642	1593	530	+2	49	20.41	GTAAA	50	58.00	CTGC
Cattle/C343R-2	KY120176	1695	45.25	RdRp	48-1652	1605	534	+3	47	25.53	GTAAA	43	44.19	CTGC
Cattle/C369R-1	KY120177	1736	42.45	RdRp	36-1721	1686	561	+3	35	22.86	GTAAA	15	40.00	CTTC
Cattle/C369R-2 ^5^	KY120179	1659	44.91	RdRp	24-1616	1593	530	NA	23	30.43	NA	43	55.81	CTGC
Cattle/C374R-1 ^5^	KY120180	1651	44.16	RdRp	8-1606	1599	532	NA	7	28.57	NA	45	55.56	CTGC
Cattle/C374R-2 ^5^	KY120181	1669	47.15	RdRp	24-1625	1602	533	NA	23	34.78	NA	44	47.73	CTGC
**Genogroup II PBVs detected in this study**														
Cattle/C372N	KY120178	1622	40.44	RdRp	55-1599	1545	514	+1	54	16.67%	GTAAA	23	52.17	CTCC

^1^ The accession number of human PBV segment 2 is NC_007027; ^2^ the accession number of human PBV segment 2 is AF246939; ^3^ the accession number of human PBV segment 2 is AF246940; ^4^ the partial segment 2 with a near complete RdRp sequence; ^5^ the partial segment 2 with complete RdRp sequences; NA, not available; T, throat swab; R, rectal swab; N, nasal swab.
